# ZEB1 regulates bone metabolism in osteoporotic rats through inducing POLDIP2 transcription

**DOI:** 10.1186/s13018-022-03312-0

**Published:** 2022-09-19

**Authors:** Xianwei Zhu, Fei Yan, Lipeng Liu, Qun Huang

**Affiliations:** 1 Department of Orthopaedics, The Affiliated Zhangjiagang Hospital of Soochow University, Suzhou, 215600 Jiangsu People’s Republic of China; 2 Department of Obstetrics and Gynecology, The Affiliated Zhangjiagang Hospital of Soochow University, No. 68, Jiyangxi Road, Zhangjiagang District, Suzhou, 215600 Jiangsu People’s Republic of China

**Keywords:** Osteoporosis, ZEB1, POLDIP2, Osteoclast, Osteoblast

## Abstract

**Background:**

Osteoporosis (OP) is a common metabolic bone disease mainly involving bone remodeling and blood vessels. The current study aimed to explore the role of zinc finger E-box binding homeobox 1 (ZEB1) in OP.

**Methods:**

First, gene expression microarrays for OP were downloaded from the Gene Expression Omnibus database and analyzed to screen for potential targets. Subsequently, a rat OP model was constructed using ovariectomy (OVX), and osteoblastic and osteoclastic differentiation and alterations in osteoporotic symptoms were observed upon intraperitoneal injection of oe-ZEB1 lentiviral vectors. DNA polymerase delta interacting protein 2 (POLDIP2) was predicted to be a downstream target of ZEB1, which was validated by ChIP-qPCR and dual-luciferase experiments. RAW264.7 cells were subjected to lentiviral vector infection of oe-ZEB1 and/or sh-POLDIP2, followed by RANKL treatment to induce osteoclast differentiation.

**Results:**

ZEB1 was poorly expressed in blood samples of postmenopausal patients with OP and in bone tissues of OVX-treated rats. Overexpression of ZEB1 or POLDIP2 in OVX rats promoted osteoblastogenesis and inhibited osteoclast differentiation. In RANKL-treated RAW264.7 cells, the transcription factor ZEB1 enhanced the expression of POLDIP2, and silencing of POLDIP2 attenuated the inhibitory effect of oe-ZEB1 on the differentiation of macrophages RAW264.7 to osteoclasts.

**Conclusions:**

ZEB1 promotes osteoblastogenesis and represses osteoclast differentiation, ultimately reducing the occurrence of postmenopausal OP by elevating the expression of POLDIP2.

## Background

Osteoporosis (OP) is a major public health burden for healthy adults over 55 years, and one in two women will develop an osteoporotic fracture compared to one in four men [1]. Contrary to the universally held misconception, bone is a relatively dynamic organ that undergoes substantial turnover compared to other organs in the body [2]. Therefore, OP results from an imbalance of the physiological process of bone turnover, with the loss of the equilibrium between the activity of osteoblasts and osteoclasts [3]. Osteoclasts degrade the bone via secretion of acid and proteolytic enzymes, such as cathepsin K (CTSK) that dissolve collagen and other matrix proteins, while osteoblasts, arising from the commitment of mesenchymal precursors through the action of transcriptional factors, produce extracellular proteins, including alkaline phosphatase (ALP) and type I collagen [4]. For people at a very high or imminent risk of fracture, management with teriparatide or abaloparatide should be considered whose duration, however, is restricted to 18–24 months [5]. Therefore, the prevention and treatment of OP have become an urgent issue to be solved.

In this study, we screened out zinc finger E-box-binding homebox 1 (ZEB1) as a significantly downregulated gene in OP using Gene Expression Omnibus (GEO) microarray. ZEB1 is a major transcription factor responsible for epithelial to mesenchymal transition and regulates cell differentiation and transformation [6]. For instance, a recent study reported that ZEB1 stimulated odontoblast differentiation in the early stage by directly binding to and increasing the enhancer activity of Dspp [7]. Therefore, we posited whether it plays a similar role in osteoblast differentiation by serving as a transcription factor. Intriguingly, DNA polymerase delta interacting protein 2 (POLDIP2) was revealed to be expressed in osteoblasts and is one of the targets of fibroblast growth factor [8]. POLDIP2 is involved in multiple protein–protein interactions and plays roles in many cellular processes, including regulation of the nuclear redox environment, pre-mRNA processing, mitochondrial morphology, cell migration and cellular adhesion [9]. RAW264.7 cells are monocyte/macrophage-like cells derived from BALB/c mice, and have become an outstanding cell tool to study osteoclast differentiation and activity due to its differentiation to osteoclast in response to receptor activator of nuclear factor kappa B ligand (RANKL) [10]. In this study, to verify the therapeutic potential of ZEB1 on OP, we examined its effect on osteoclast differentiation and osteogenesis and elucidated the molecular mechanism in RAW264.7 cells exposed to RANKL. In addition, ovariectomy (OVX)-induced OP rats were used to evaluate the possible alleviating effects of ZEB1 and POLDIP2 on OP.

## Materials and methods

### Clinical samples

This study was permitted by the Ethic Board of the Affiliated Zhangjiagang Hospital of Soochow University. All participants signed the written informed consent. The experiments were carried out according to the *Declaration of Helsinki*. Forty postmenopausal patients with OP and 40 postmenopausal women with normal bone mass (normal) were enrolled in this study between September 2018 and May 2020 from the Affiliated Zhangjiagang Hospital of Soochow University. All samples were diagnosed by dual-energy X-ray absorptiometry according to the criteria established by the World Health Organization. According to the Guidelines for the diagnosis and management of primary OP, patients with a bone strength value T-score < − 2.5 were diagnosed as OP and they had not received any treatment within 3 months prior to admission. Detailed information of OP patients and normal controls is shown in Table 1. In addition, we excluded patients with other clinical comorbidities, such as cancer, diabetes, cardiovascular disease, inflammation and metabolic disorders.

**Table 1:** Characteristics of participants recruited for the study

	Normal	OP patients	*p* value
Age (years)	61.85 ± 6.21	62.26 ± 5.85	NS
Postmenopausal (years)	8.15 ± 2.37	8.62 ± 3.09	NS
BMI (kg/m^2^)	23.67 ± 3.15	21.23 ± 2.76	NS
Spine T-score	0.78 ± 0.21	− 3.61 ± 0.36	< 0.01
Femoral neck T-score	0.96 ± 0.35	− 2.37 ± 0.58	< 0.01

NS, not significant; OP, osteoporosis; BMI, body mass index

### Ovariectomy (OVX)-induced OP rat model

All rat experiments were reviewed and permitted by the Institutional Animal Care and Use Committee of the Laboratory Animal Center of the Affiliated Zhangjiagang Hospital of Soochow University. Thirty 8-week-old female Sprague–Dawley rats (Shanghai Institutes for Biological Sciences, Chinese Academy of Sciences, Shanghai, China) were housed in specific pathogen-free-grade cages on a 12-h light and 12-h dark cycle. After one week of acclimatization, the rats were randomly allocated into five groups: sham-operated group (Sham group), OVX group, OVX + overexpression (oe)-negative control (NC) injection group (oe-NC group), OVX + oe-ZEB1 injection group (oe-ZEB1 group), and OVX + oe-POLDIP2 injection group (oe-POLDIP2 group). The rats were anesthetized by the administration of 1% pentobarbital sodium (45 mg/kg, *i.p.*), and bilateral OVX was performed to develop an OP model [11]. Oe-NC, oe-ZEB1, oe-POLDIP2 (all at 7 ng/kg) were injected into ovariectomized rats intraperitoneally on the day 1–3, 15–18, and 29–32 for a total of 9 injections. The sham group comprised rats whose ovaries were exposed but not removed. After five weeks, the rats were euthanized. The femurs were separated, fixed in 4% paraformaldehyde solution, and stored for subsequent studies.

### Cell culture and treatment

Well-grown murine macrophages RAW264.7 (SC-6003, American Type Culture Collection, Manassas, VA, USA) were infected with the constructed lentiviral vectors harboring oe-NC, oe-ZEB1, oe-POLDIP2 or sh-POLDIP2 (TTGGACTGACCTGACTATAAA) (all from VectorBuilder, Guangzhou, Guangdong, China).

The RAW264.7 cells were cultured in the presence of 50 ng/mL RANKL (R&D Systems Inc., Minneapolis, MN, USA) or control phosphate-buffered saline (PBS) for 3 d at 37 °C and 5% CO_2_.

### Reverse transcription-quantitative PCR (RT-qPCR)

Total RNA was extracted from plasma using RNAzol® RT RNA Isolation Reagent (Sigma-Aldrich Chemical Company, St Louis, MO, USA). Total RNA was isolated from tissues or cells using TRIzol (Invitrogen, Carlsbad, CA, USA) by referring to the manufacturer's instructions. Isolated RNA was quantified by NanoDro-pND-2000 spectrophotometer (Thermo Fisher Scientific). cDNA was obtained by RNA reverse transcription using the Quanti-Tect Reverse Transcription Kit (Qiagen company, Hilden, Germany). PCR of transcription products was performed by SYBR Green PCR Master Mix (Applied Biosystems, Inc., Foster City, CA, USA) on a CFX96 Touch Real-Time PCR Detection System (Bio-Rad Laboratories, Hercules, CA, USA). The expression of target genes was calculated using the 2^−ΔΔCt^ method and normalized to the reference gene glyceraldehyde-3-phosphate dehydrogenase (GAPDH). Table 2 shows the detailed sequences of the specific primers (Shanghai Sangon Biological Engineering Technology & Services Co., Ltd., Shanghai, China).

**Table 2:** Primer sequences

Name of primer	Sequences (5′−3′)
hsa-ZEB1-F	GGCATACACCTACTCAACTACGG
hsa-ZEB1-R	TGGGCGGTGTAGAATCAGAGTC
hsa-POLDIP2-F	TACCGAGGTGTCGTCCTGTTTC
hsa-POLDIP2-R	CCTTTCACCTCCTTGGAGCCAT
rno-ZEB1-F	CGGCGCAATAACGTTACAAAT
rno-ZEB1-R	CACTGTCTGGTCTGTTGGCA
rno-POLDIP2-F	ATCCAGACCACAAGTGCAGG
rno-POLDIP2-R	GGAACCCTAAGAACTGGGGC
rno-ALP-F	CCTTAGGGCCACCGCTCG
rno-ALP-R	GTTCAGTGCGGTTCCAGACA
rno-OPN-F	CCAGCCAAGGACCAACTACA
rno-OPN-R	GAACTCGCCTGACTGTCGAT
rno-RUNX2-F	CCTGAACTCTGCACCAAGTCCT
rno-RUNX2-R	CATCTGGCTCAGATAGGAGGG
rno-NFATC-F	GGTGCCTTTTGCGAGCAGTATC
rno-NFATC-R	CGTATGGACCAGAATGTGACGG
rno-CTSK-F	AGCAGAACGGAGGCATTGACTC
rno-CTSK-R	CCCTCTGCATTTAGCTGCCTTTG
rno-GAPDH-F	CATCACTGCCACCCAGAAGACTG
rno-GAPDH-R	ATGCCAGTGAGCTTCCCGTTCAG
rno-β-actin-F	CATTGCTGACAGGATGCAGAAGG
rno-β-actin-R	TGCTGGAAGGTGGACAGTGAGG
hsa-β-actin-F	CACCATTGGCAATGAGCGGTTC
hsa-β-actin-R	AGGTCTTTGCGGATGTCCACGT
hsa-GAPDH-F	GTCTCCTCTGACTTCAACAGCG
hsa-GAPDH-R	ACCACCCTGTTGCTGTAGCCAA
rno-ALDH3B1-F	TTGGCGAGGCCTCAGAATG
rno-ALDH3B1-R	CTCAGCCTGACTGATGGCAA
rno-PAF1-F	GAAAGCGGGACCAAGAGGAA
rno-PAF1-R	GGACCCTGGTCTCTAGCTCA
rno-ITGAL-F	CCCAGGCTGCAGTTATCACA
rno-ITGAL-R	AACGCATGCCCTAACCTCAA
rno-TSR3-F	GGTGAGTTTGGAGCAGTCGG
rno-TSR3-R	CCAAGCTCCCACATAGCCAA
rno-NADK-F	TGCAGGGCGATGATTATGCT
rno-NADK-R	ACTGAGCAAGGCTCTCGAAC
mmu-ZEB1-F	ATTCAGCTACTGTGAGCCCTGC
mmu-ZEB1-R	CATTCTGGTCCTCCACAGTGGA
mmu-POLDIP2-F	AGTGCTGGAGACAGTTGGTGTG
mmu-POLDIP2-R	CTGGAGTTGCAGAAGCCACATC
mmu-NFATC-F	GGTGCCTTTTGCGAGCAGTATC
mmu-NFATC-R	CGTATGGACCAGAATGTGACGG
mmu-CTSK-F	AGCAGAACGGAGGCATTGACTC
mmu-CTSK-R	CCCTCTGCATTTAGCTGCCTTTG
mmu-GAPDH-F	CATCACTGCCACCCAGAAGACTG
mmu-GAPDH-R	ATGCCAGTGAGCTTCCCGTTCAG
mmu-POLDIP2 Promoter-F	GCGGAGTTGCGTCTGTATTT
mmu-POLDIP2 Promoter-R	ACACAGACATCTCCACAGCA

hsa, homo sapiens; rno, rattus norvegicus; mmu, mus musculus; ZEB1, zinc finger E-box binding homeobox 1; POLDIP2, DNA polymerase delta interacting protein 2; ALP, alkaline phosphatase; OPN, osteopontin; RUNX2, Runt-related transcription factor 2; NFATC, nuclear factor of activated T cells transcription complex; CTSK, cathepsin K; GAPDH, glyceraldehyde-3-phosphate dehydrogenase; ALDH3B1, aldehyde dehydrogenase family 3 member B1; PAF1, RNA polymerase II-associated protein 1; ITGAL, Integrin alpha-L; TSR3, 18S rRNA aminocarboxypropyltransferase; NADK, NAD kinase; F, forward; R, reverse

### Western blot

Total protein was extracted from cells and tissues by radio immunoprecipitation assay buffer (Sigma) containing a mixture of protease and phosphatase inhibitors (Beyotime, Shanghai, China), and total protein was determined using a Micro BCA™ Protein Assay kit (Thermo Fisher Scientific, Inc.). Then, 30 µg protein sample was separated by 12% sodium dodecyl sulfate–polyacrylamide gels and transferred to polyvinylidene fluoride membranes (Millipore, Billerica, MA, USA). The membranes were sealed with 5% BSA (Beyotime) for 1 h at room temperature, followed by an overnight incubation with primary antibodies against GAPDH (1:10,000, GTX100118, GeneTex, Inc., Alton Pkwy Irvine, CA, USA), ZEB1 (1:1000, GTX33589, GeneTex), ALP (1:2000, ab154100, Abcam, Cambridge, MA, USA), OPN (1:1000, ab63856, Abcam), RUNX2 (1:1000, GTX00792, GeneTex), MMP9 (1:1000, ab283575, Abcam), c-Fos (1:2000. GTX129846, GeneTex), Acp5 (1:1000, PA5-116970, Thermo Fisher Scientific), OPG (1:1000, GTX55734, GeneTex), NFATC (1:2000, GTX22796, GeneTex) and CTSK (1:1000, ab187647, Abcam) at 4 °C. Horseradish peroxidase-conjugated secondary antibody to IgG (1:5000, ab6721, Abcam) was incubated with the membrane for 2 h at room temperature. The membranes were developed using an enhanced chemiluminescent (Beyotime) reagent. The intensity of each protein band was assessed using ImageJ (National Institutes of Health, Bethesda, MD, USA).

### Immunohistochemistry

Immunohistochemical staining was performed to evaluate the expression of osteoblast marker OPG and osteoclast marker TRAP. Rat femurs were fixed with 4% paraformaldehyde, embedded in paraffin, sectioned at 5 μm, and routinely dewaxed. Tissue sections were treated with citric acid antigen repair buffer (pH = 6.0) for antigen retrieval. After natural cooling, the sections were placed in PBS (pH = 7.4) and shaken on a decolorization shaker. Endogenous peroxidase was quenched with 3% hydrogen peroxide for 10 min. The sections were incubated with primary antibodies to TRAP (1:500, ab65854, Abcam) and OPG (1:200, GTX55734, GeneTex) overnight at 4 °C and with goat anti-rabbit IgG secondary antibody (1:1000, ab150077, Abcam) for 1 h at room temperature. The color was developed using diaminobenzidine (Merck Millipore, Darmstadt, Germany), and the nuclei were stained using hematoxylin (Sigma-Aldrich). After being sealed with neutral gum, the sections were observed under a microscope (Nikon, Tokyo, Japan). Positive cell rates were calculated by ImageJ (Image J v46a, NIH) software.

### Hematoxylin–eosin (HE) staining

The femur was fixed in 10% formalin buffer, decalcified with 10% ethylenediaminetetraacetic acid for 4 weeks, and then embedded in paraffin. The samples were cut in half (longitudinally in the sagittal plane) to standardize the sections of each specimen at the sagittal midplane, and the femoral tissues were cut into 5-μm sections along the long axis of each femur in the sagittal plane using a rotary sectioning machine (Thermo Fisher) and then embedded in paraffin. In strict accordance with the instructions of the HE staining kit (Beyotime), the sections were dewaxed with xylene, hydrated with gradient ethanol, stained with hematoxylin for 5 min, and put into 1% hydrochloric acid alcohol for 2 s. The sections were sealed after dipping into gradient alcohol again for 2 min and eosin staining for 1 min, and viewed under a microscope (Nikon) to observe structural changes in bone tissues.

### Chromatin immunoprecipitation (ChIP)

ChIP analysis was performed using a ChIP kit (Millipore). The cells were fixed using 4% paraformaldehyde for 10 min at room temperature, and DNA was sonicated until the sheared DNA was ~ 200–1000 bp in size. The supernatant was harvested by centrifugation at 13,000 rpm for 5 min at 4 °C, and the chromatin was incubated overnight with the antibody against ZEB1 (ab155249, Abcam). After immunoprecipitation of the cell lysates overnight, qPCR was used to analyze the enrichment of the POLDIP2 promoter.

### Luciferase reporter assay

We obtained the rat POLDIP2 promoter (range = chr10: 65,780,765–65,781,764) sequence from NCBI (https://www.ncbi.nlm.nih.gov/). The potential binding sequence of this POLDIP2 promoter sequence to ZEB1 was obtained from JASPAR (http://jaspar.genereg.net/). The binding region was amplified using PCR and cloned into the pGL3 vector (Promega Corporation, Madison, WI, USA) to construct a luciferase reporter vector for Promoter. The Promoter luciferase reporter vector was co-transfected into cells with oe-NC or oe-ZEB1 using Lipofectamine 2000 (Thermo Fisher Scientific). Forty-eight hours after transfection, luciferase activity was tested by Dual-luciferase reporter assay system (Promega).

### Data analysis

The data were displayed as mean ± standard deviation (SD). The analyses were performed in SPSS ver. 22.0 (IBM Corp. Armonk, N.Y., USA). Each experiment in vitro was repeated three times. Differences were tested using unpaired t-test (between two groups) and two-way analysis of variance (ANOVA); the Tukey’ post hoc test was applied to compare three or more groups. *p < 0.05, **p < 0.01, and ***p < 0.001 were considered indicative of statistical significance.

## Results

### ZEB1 is poorly expressed in postmenopausal osteoporotic patients and OVX rats

**Fig. 1:**
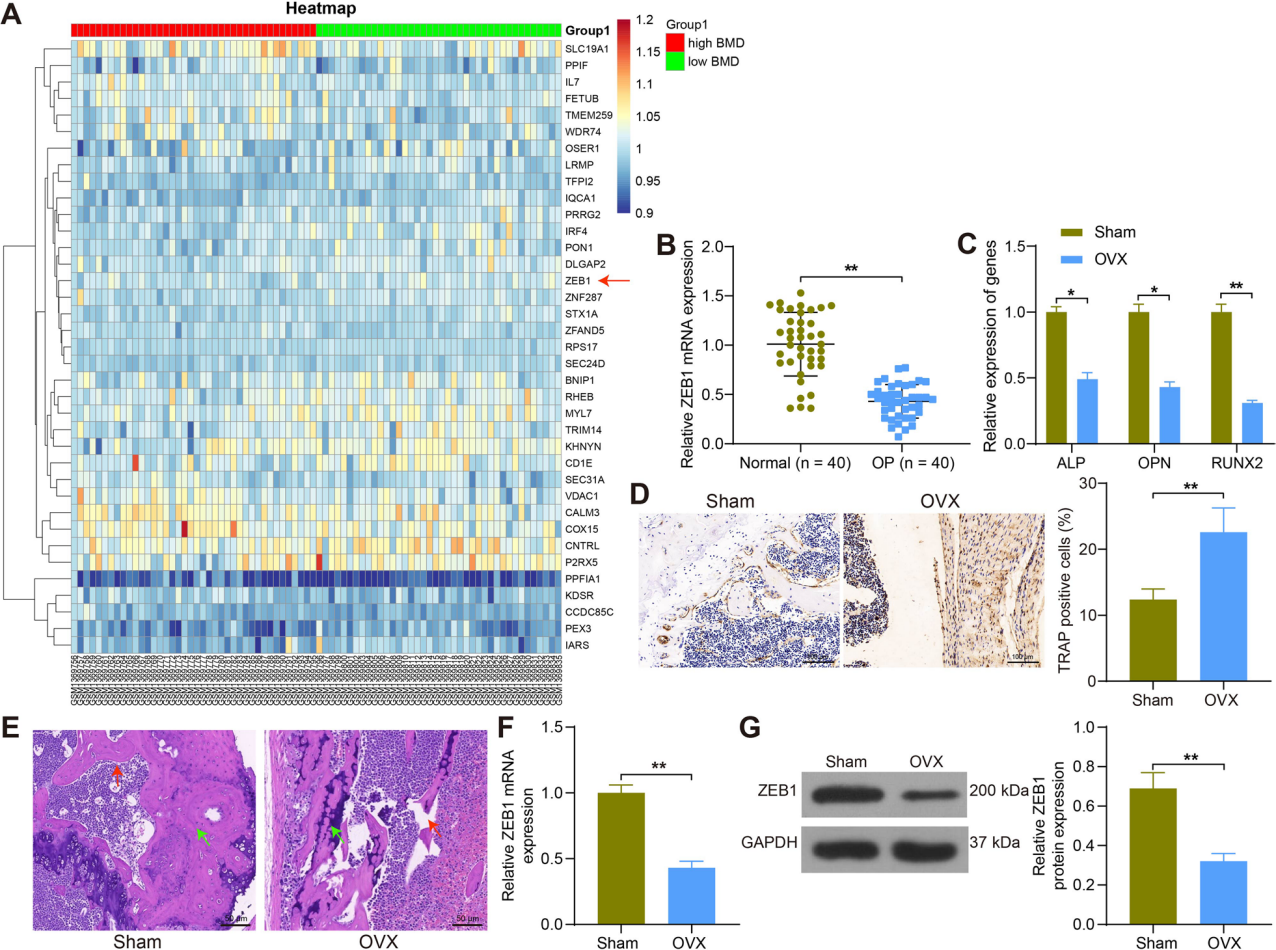
ZEB1 was poorly expressed in both postmenopausal osteoporotic patients and rats. **A** The heatmap analysis of differentially expressed genes in the GSE56815 dataset where BMD refers to bone mineral density. **B** Detection of ZEB1 expression in the blood of normal controls and OP patients by RT-qPCR (n = 40). **C** Detection of ALP, OPN and RUNX2 expression in bone tissues of sham- and OVX-treated rat by RT-qPCR. **D** Detection of TRAP expression in rat bone tissue by immunohistochemistry. **E** Assessment of bone content and structural changes in femoral tissues of rats by HE staining where bone trabeculae were labeled with red arrows and bone mass was labeled with green arrows. **F**–**G** Expression of ZEB1 in rat bone tissues at mRNA and protein levels by RT-qPCR (**F**) and Western blot (**G**). All data are represented as mean ± SD (n = 6 for rats in each group). Each experiment was performed three times independently. *p < 0.05, **p < 0.01. Differences are tested using two-way ANOVA with Tukey’s post hoc test (**C**) and unpaired *t*-test (**B**, **D**, **F**, **G**)

First, we downloaded the OP-related microarray GSE56815 from the GEO database, which included 40 premenopausal high bone density samples and 40 postmenopausal low bone density samples and screened for differentially expressed genes by setting the screening conditions of|Log FC|> 1 and Adj *p* value < 0.05 (Fig. 1A). ZEB1, which showed the largest alteration and was rarely studied, was chosen. Then, we analyzed the collected blood samples by RT-qPCR and found that ZEB1 was significantly poorly expressed in postmenopausal patients with OP (Fig. 1B). Meanwhile, we constructed an OP rat model by OVX. Bone formation markers ALP, OPN and RUNX2 were detected by RT-qPCR in femoral tissues of rats subjected to OVX. It was observed that the expression of all these markers was significantly reduced, and the bone formation of rats was diminished (Fig. 1C). Subsequently, we used immunohistochemistry to detect the osteoclast marker protein TRAP in bone tissues, and the results showed that TRAP was significantly elevated in the bone tissues of OVX rats, with significant osteoclast aggregation (Fig. 1D). HE staining revealed that OVX rats had reduced bone mass, significant bone loss and damaged trabecular microstructure (Fig. 1E). These observations indicated that the OP rat model was successfully developed. Moreover, the results of RT-qPCR and Western blot assay showed that ZEB1 was significantly downregulated in the bone tissues of osteoporotic rats (Fig. 1F, G).

### ZEB1 promotes osteoblast formation and inhibits osteoclast differentiation

**Fig. 2:**
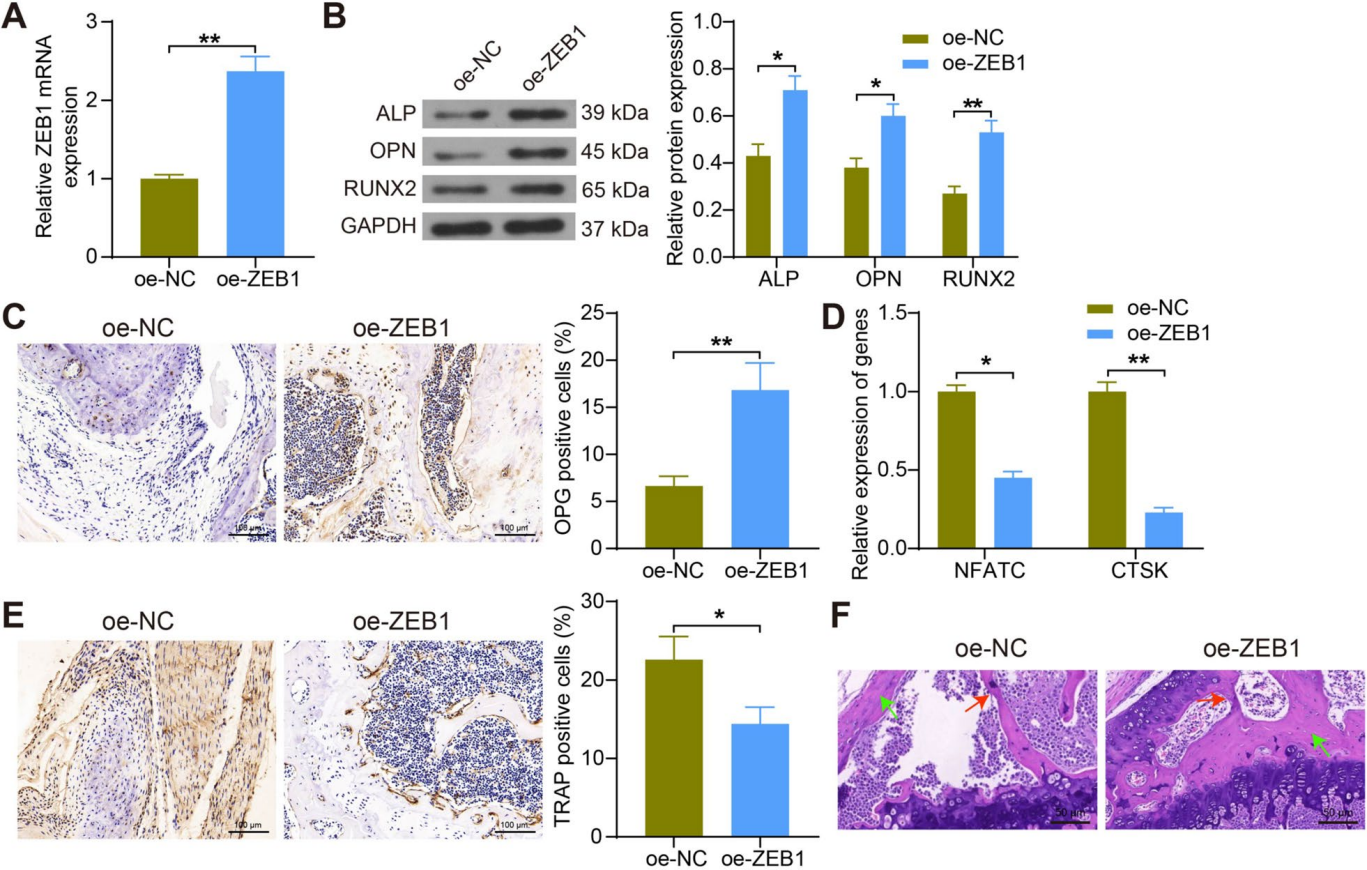
ZEB1 supports osteoblast differentiation and restrains osteoclast differentiation. **A** Detection of mRNA expression of ZEB1 in bone tissues of rats in response to oe-NC or oe-ZEB1 by RT-qPCR. **B** Detection of protein expression of ALP, OPN and RUNX2 in bone tissues of rats by Western blot. **C** Immunohistochemical detection of OPG expression in bone tissues of rats. **D** Detection of mRNA expression of NFATC and CTSK in bone tissues of rats by RT-qPCR. **E** Immunohistochemical detection of TRAP expression in bone tissues of rats. **F** Assessment of bone content and structural changes in rat bone tissue by HE staining where bone trabeculae were labeled with red arrows and bone mass was labeled with green arrows. All data are represented as mean ± SD (*n* = 6 for rats in each group). Each experiment was performed three times independently. *p < 0.05, **p < 0.01. Differences are tested using two-way ANOVA with Tukey’s post hoc test (**B**, **D**) and unpaired t-test (**A**, **C**, **E**)

To further clarify the role played by ZEB1 in OP, we constructed a lentiviral vector for oe-ZEB1 and injected it into OVX rats intraperitoneally. The expression of ZEB1 in the bone tissues of the rats in the oe-ZEB1 group was significantly increased using RT-qPCR (Fig. 2A). The protein expression of ALP, OPN and RUNX2 in the bone tissues of rats in the oe-ZEB1 group was observed to be appreciably enhanced using Western blot assay (Fig. 2B). Immunohistochemical detection demonstrated that the expression of OPG was elevated in the bone tissues of rats injected with oe-ZEB1 (Fig. 2C). Detection of osteoclast markers NFATC and CTSK in rat bone tissues using RT-qPCR showed that both mRNA expression was significantly reduced (Fig. 2D). Immunohistochemical assay revealed that TRAP expression was inhibited in the bone tissues of rats treated with oe-ZEB1 (Fig. 2E). HE staining of rat bone tissues showed a significant increase in bone volume, reduced bone loss and partial restoration of trabecular damaged microstructure in the oe-ZEB1 group compared to the oe-NC group (Fig. 2F). These findings above suggest that ZEB1 promotes osteoblast formation and inhibits osteoclast differentiation in osteoporotic rats.

### ZEB1 promotes the transcription of POLDIP2

**Fig. 3:**
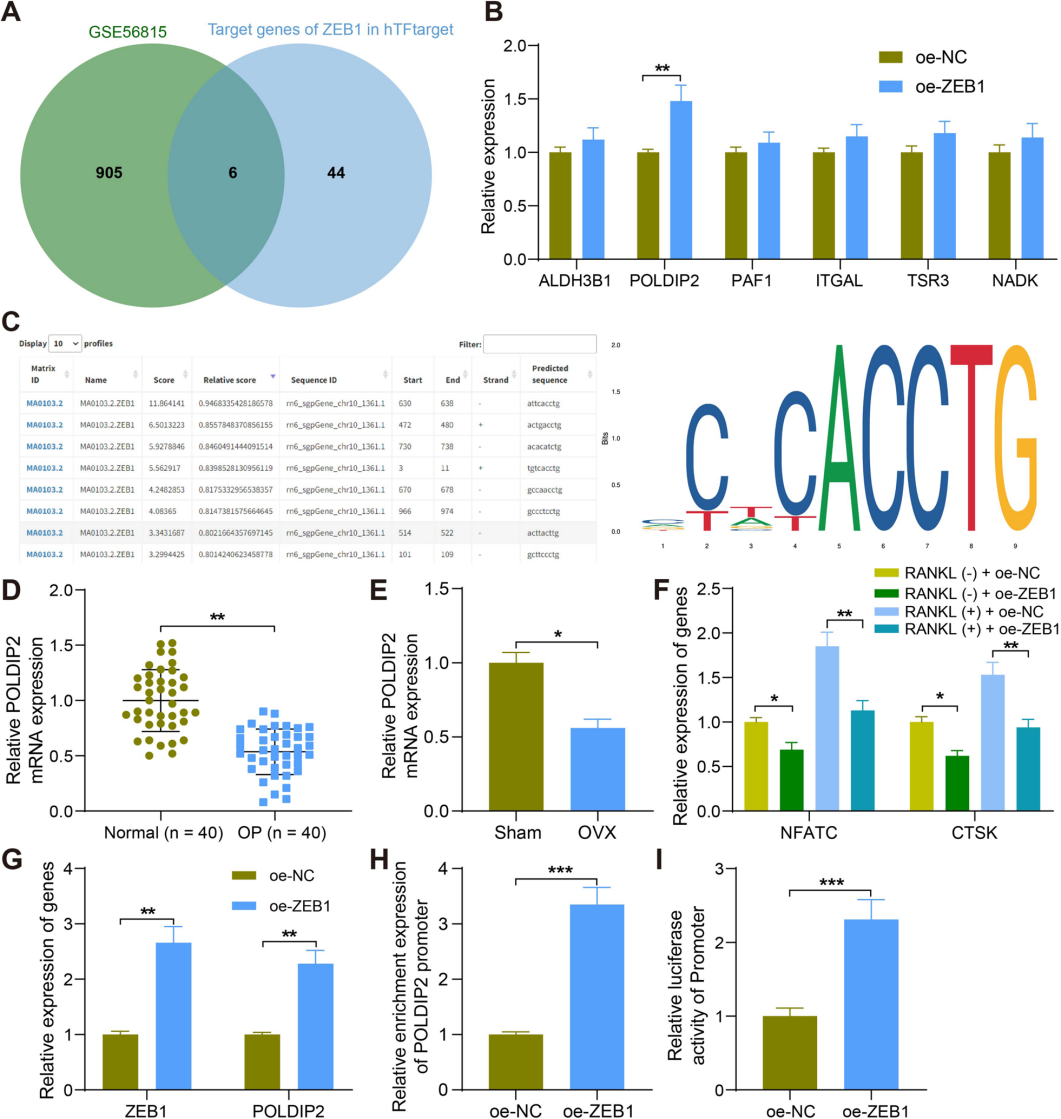
ZEB1 promotes the transcription of POLDIP2. **A** The intersection of top 50 downstream targets of ZEB1 in the hTFtarget database and significantly differentially expressed genes screened in the GSE56815 dataset. **B** Detection of expression changes of six intersecting genes in bone tissues of rats overexpressing ZEB1. **C** Conserved binding sites for ZEB1 predicted using JASPAR and potential binding sites of ZEB1 to POLDIP2 promoter. **D** POLDIP2 expression in the blood of normal controls and OP patients (*n* = 40). **E** mRNA expression of POLDIP2 in bone tissues of sham- and OVX-treated rats by RT-qPCR. **F** mRNA expression of NFATC and CTSK in RAW264.7 cells infected with oe-NC or oe-ZEB1 after RANKL treatment by RT-qPCR. **G** Detection of mRNA expression of ZEB1 and POLDIP2 in RAW264.7 cells in response to oe-NC or oe-ZEB1 by RT-qPCR. **H** The enrichment ability of Anti-ZEB1 on POLDIP2 promoter in RAW264.7 cells in response to oe-NC or oe-ZEB1 by ChIP-qPCR. **I** Promoter luciferase activity of POLDIP2 examined in RAW264.7 cells in response to oe-NC or oe-ZEB1 using dual-luciferase assay. All data are represented as mean ± SD (*n* = 6 for rats in each group). Each experiment was performed three times independently. *p < 0.05, **p < 0.01, ***p < 0.001. Differences are tested using two-way ANOVA with Tukey’s post hoc test (**F**, **G**) and unpaired *t*-test (**D**, **E**, **H**, **I**)

We then focused on the downstream target of ZEB1 and predicted 50 candidates in the hTFtarget database (http://bioinfo.life.hust.edu.cn/hTFtarget#!/tf). The predicted 50 targets were intersected with 911 differentially expressed genes in the GSE56815 microarray. The results showed a total of six intersecting genes: ALDH3B1, POLDIP2, PAF1, ITGAL, TSR3, and NADK (Fig. 3A). We examined changes in the expression of six intersecting genes in the bone tissues of rats overexpressing ZEB1. Only POLDIP2 expression was significantly increased (Fig. 3B). Therefore, we selected POLDIP2 for our following study.

According to the conserved binding site of ZEB1 obtained from the JASPAR website, and the potential binding site of POLDIP2 to ZEB1 (Fig. 3C) was predicted. We found significant loss of POLDIP2 expression in blood samples from postmenopausal patients with OP and in OVX rats by RT-qPCR analysis (Fig. 3D, E). Then, RAW264.7 cells were delivered with oe-NC or oe-ZEB1 vectors, followed by RANKL treatment to prompt their differentiation towards osteoclasts. The mRNA expression of NFATC and CTSK was found to be significantly elevated after treatment with RANKL by RT-qPCR, which was reduced by the overexpression of ZEB1 (Fig. 3F). The mRNA expression of both ZEB1 and POLDIP2 was significantly elevated in the oe-ZEB1 group (Fig. 3G). ChIP and dual-luciferase reporter assays were used to explore the relationship between POLDIP2 and ZEB1. After ZEB1 upregulation, the enrichment of anti-ZEB1 in the promoter region of POLDIP2 was significantly increased (Fig. 3H), and the luciferase activity of POLDIP2 (Fig. 3I) was enhanced. The results suggest that ZEB1 can target to POLDIP2 and promote its expression.

### POLDIP2 attenuates OP in rats by regulating osteoclast differentiation

**Fig. 4:**
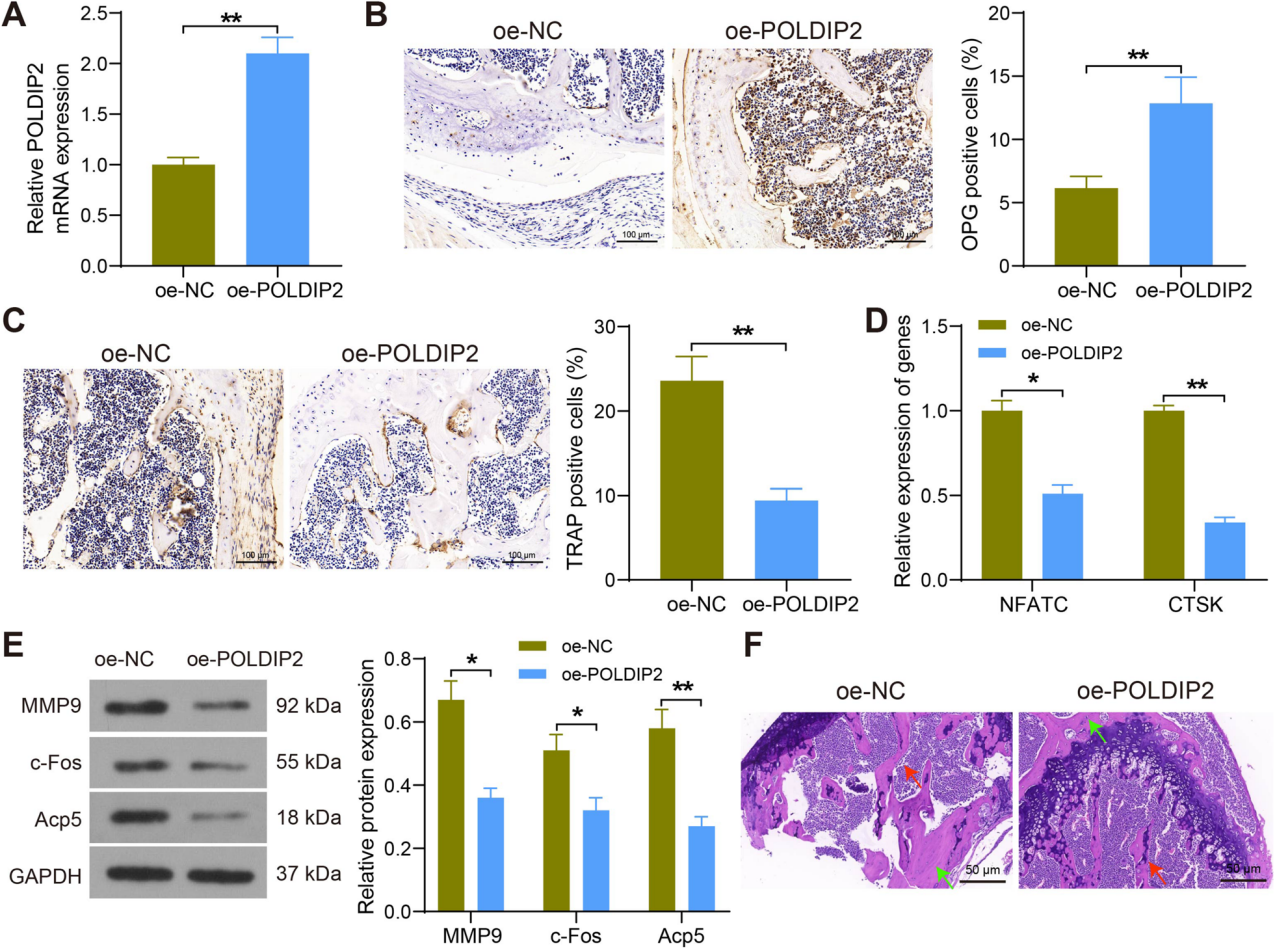
POLDIP2 attenuates OP in rats by regulating osteoclast differentiation. **A** Detection of mRNA expression of POLDIP2 in bone tissues of rats by RT-qPCR. **B**, **C** Immunohistochemical detection of OPG (**B**) and TRAP **C** expression in bone tissues of rats. **D** Detection of mRNA expression of NFATC and CTSK in bone tissues of rats by RT-qPCR. **E** Detection of protein expression of MMP9, c-Fos and Acp5 in bone tissue of rats by Western blot. **F** Assessment of bone content and structural changes in bone tissue of rats by HE staining where bone trabeculae were labeled with red arrows and bone mass was labeled with green arrows. All data are represented as mean ± SD (*n* = 6 for rats in each group). Each experiment was performed three times independently. *p < 0.05, **p < 0.01. Differences are tested using two-way ANOVA with Tukey’s post hoc test (**D**, **E**) and unpaired *t*-test (**A**–**C**)

The lentiviral vector for oe-POLDIP2 was injected intraperitoneally into OVX rats. The mRNA expression of POLDIP2 was significantly increased in the bone tissues of rats in the oe-POLDIP2 group, as revealed by RT-qPCR (Fig. 4A). Immunohistochemical assays revealed that the expression of the osteoblast marker OPG was significantly augmented (Fig. 4B) and the expression of the osteoclast marker TRAP was decreased (Fig. 4C) in the bone tissues of rats in the oe-POLDIP2 group. The mRNA expression of the osteoclast markers NFATC and CTSK in the bone tissues of OVX rats was drastically reduced using RT-qPCR assay after oe-POLDIP2 injection (Fig. 4D). Subsequently, the protein expression of MMP9, c-Fos and Acp5 in rat bone tissues was further measured using Western blot, and we observed that osteoclastogenesis was significantly inhibited after oe-POLDIP2 (Fig. 4E). The HE staining results showed a significant increase in bone volume, significant preservation of bone mass and reduced damage to trabecular structures in the oe-POLDIP2-treated rats (Fig. 4F). Taken together, these results indicated that POLDIP2 can attenuate OP by regulating the differentiation of osteoclasts.

### Silencing of POLDIP2 mitigates the inhibitory effect of oe-ZEB1 on the differentiation of RAW264.7 to osteoclasts

**Fig. 5:**
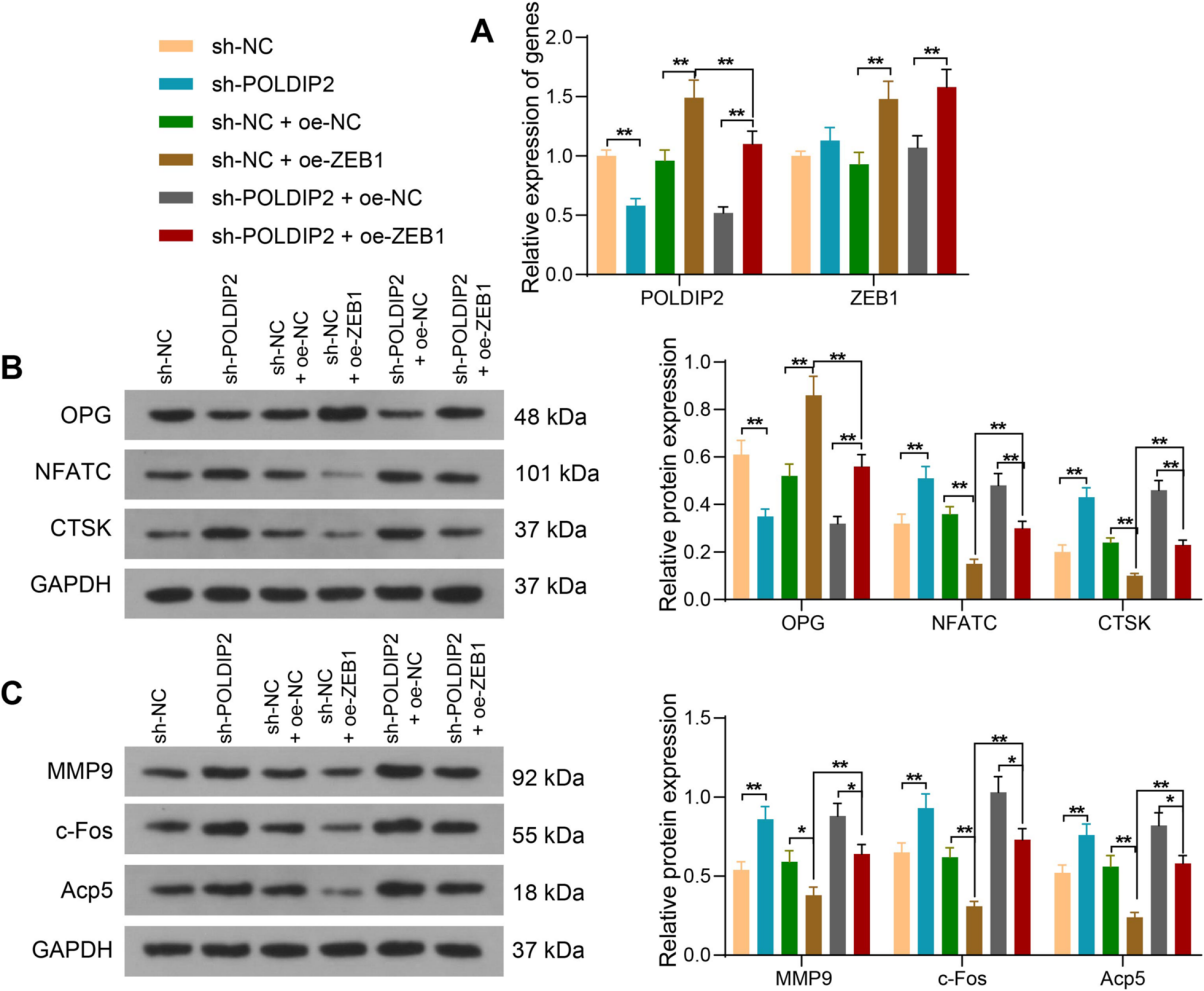
Silencing of POLDIP2 attenuates the inhibitory effect of oe-ZEB1 on the differentiation of macrophages RAW264.7 to osteoclasts. RAW264.7 cells were infected with sh-NC, sh-POLDIP2, sh-NC + oe-NC, sh-NC + oe-ZEB1, sh-POLDIP2 + oe-NC or sh-POLDIP2 + oe-ZEB1. **A** mRNA expression of POLDIP2 and ZEB1 in cells after infection by RT-qPCR. **B** Detection of protein expression of OPG, NFATC and CTSK in cells after infection by Western blot analysis. **C** The protein expression of MMP9, c-Fos and Acp5 in cells after infection detected by Western blot analysis. All data are represented as mean ± SD. Each experiment was performed three times independently. *p < 0.05, **p < 0.01. Differences are tested using two-way ANOVA with Tukey’s post hoc test

Macrophages RAW264.7 were infected with sh-POLDIP2 alone (sh-NC as control), sh-NC + oe-ZEB1 (sh-NC + oe-NC as control) or sh-POLDIP2 + oe-ZEB1 (sh-POLDIP2 + oe-NC). RT-qPCR assay was used to determine the overexpression or knockdown efficacy (Fig. 5A). After further treatment of RAW264.7 cells with RANKL, we observed that protein expression of OPG was decreased, and protein expression of both NFATC and CTSK was enhanced after silencing of POLDIP2. However, the overexpression of ZEB1 contributed to the opposite trends and reversed the inhibiting effects of sh-POLDIP2 on OPG levels and stimulating effects on NFATC and CTSK levels (Fig. 5B). Moreover, the protein expression of MMP9, c-Fos and Acp5 was found to increase after silencing of POLDIP2. Their expression was found to be significantly decreased by upregulation of ZEB1, which was reversed by silencing of POLDIP2 (Fig. 5C). In summary, the transcription factor ZEB1 can support osteoblast differentiation and inhibit osteoclast differentiation by mediating POLDIP2 to reduce the occurrence of postmenopausal OP.

## Discussion

Postmenopausal OP affects millions of females since estrogen deficiency is the main factor in the pathogenesis of OP [12]. The balance between the activities of bone-forming osteoblasts and bone-resorbing osteoclasts is of great importance to the strength and integrity of the bone [13]. With aging, the balance has a high propensity for bone resorption during which the osteoclast activity exceeds the osteoblast activity, causing fracture [14]. Therefore, unveiling the mechanism underlying the balance between osteoclast and osteoblasts might have therapeutic potential for OP. Here, we showed that ZEB1 is poorly expressed in the blood samples of patients with OP and OVX rats. In addition, ZEB1 promoted the expression of POLDIP2 transcriptionally to elevate the osteoblast formation and suppress osteoclast differentiation, thus alleviating OP in rats with OVX.

With the help of microarray analysis, we identified ZEB1 as one of the most downregulated genes in OP, which was further validated in our collected clinical blood samples and bone tissues from OVX rats. The expression of ZEB1 has been assessed in embryos from chick, mouse, and to a more limited degree in adult human, mouse, and rat tissues [15]. As a driver of epithelial-mesenchymal transition, ZEB1 contributed to tumor progression and metastasis and correlated to dismal clinical outcomes in cancer patients [16]. More relevantly, it has been reported that endothelial ZEB1 deletion impaired bone formation [17]. In addition, ZEB1 overexpression enhanced the expression of COL1A1, RUNX2, and OCN in bone mesenchymal stem cells [18]. Osteoblast differentiation is governed by the master transcription factor RUNX2, and RUNX2 null mice have a completely lack mineralized tissues because of osteoblast maturation termination [19]. NFATc1, c-Fos and TRAP are major transcription factors during osteoclast differentiation, and dysregulation of these genes may cause osteoclastogenesis [20]. In the present study, we observed that ALP, OPN, RUNX2, and OPG (all osteoblast markers) were upregulated, while NFATC, CTSK, and TRAP (all osteoclast markers) were downregulated in bone tissues of rats injected with oe-ZEB1. Therefore, we innovatively established the suppressive effects of oe-ZEB1 on osteoclast differentiation. β-catenin-coordinated long non-coding RNA MALAT1/microRNA-217 axis could enhance expression of ZEB1 and promote the differentiation of bone marrow mesenchymal stem cells into hepatocytes [21]. However, few studies focused on the upstream mechanism of ZEB1 in OP. Interestingly, ZEB1 has been revealed to be normally regulated by both estrogen and progesterone receptors [22], which might be responsible for the downregulation of ZEB1 under the condition of postmenopausal OP where estrogen deficiency is frequently seen.

Subsequently, we identified POLDIP2 as a direct target of ZEB1 in OP. POLDIP2 is an understudied protein, originally defined as a binding partner of polymerase delta and proliferating cell nuclear antigen [23]. POLDIP2 (also called PDIP38) has a number of other functions, including roles in organizing the mitotic spindle, DNA repair, and cellular adhesion [24, 25]. POLDIP2 has been identified as a mediator of cell metabolism and mitochondrial function that involves in metabolic adaptation [26]. Amanso et al*.* revealed that POLDIP2 promoted ischemia-induced collateral vessel formation via various mechanisms that likely involve reactive oxygen species-dependent induction of matrix metalloproteinase activity, and augmented vascular cell growth and survival [27]. However, its participation in bone-related disorders remains to be explored. Indeed, POLDIP2 was expressed in femoral bone in vivo and its levels were enhanced in aged mice compared to young adult mice [8]. Downregulation of POLDIP2 via shPOLDIP2 markedly inhibited lipopolysaccharide-induced increases in the expression of Nox4 [28], which was upregulated in differentiated osteoclasts compared to osteoclast precursors, indicating its role in the physiology and pathobiology of bone and joints [29]. We found here that TRAP, NFATC, CTSK, MMP9, c-Fos, and Acp5 expression was significantly downregulated in bone tissues of OVX rats following ectopic expression of POLDIP2. More importantly, silencing of POLDIP2 reversed the inhibitory effects of ZEB1 on the differentiation of RAW264.7 cells. The paradoxical function has yet to be elucidated and might be partly attributed to a multitude of mechanisms of OP pathobiology. It has been demonstrated that RANKL can increase the expression of Acp5 through NFATC and c-Fos to accelerate osteoclastogenesis, which also can be modulated by many contributors [30]. In this present study, we observed that ZEB1-regulated POLDIP2 might involve in this process.

## Conclusion

Using in vivo OVX rats and in vitro RAW264.7 cell cultures, we show that ZEB1 induces bone formation and represses osteoclast differentiation by increasing the expression of POLDIP2. The present study provides further information about the molecular mechanism of bone remodeling, and will thus help in future potential therapeutic studies on postmenopausal OP. However, this study is still a preliminary one, and we cannot exclude the involvement of other biomolecules in the regulation of osteoblast and osteoclast activities due to the complex microenvironments. Thus, more in-depth investigations are warranted.

## Data Availability

The data used to support the findings of this study are available from the corresponding author upon request.

## Abbreviations

OP: Osteoporosis
ZEB1: Zinc finger E-box binding homeobox 1
POLDIP2: DNA polymerase delta interacting protein 2
OVX: Ovariectomy
PBS: Phosphate-buffered saline
GAPDH: Glyceraldehyde-3-phosphate dehydrogenase
BSA: Bovine serum albumin
HE: Hematoxylin-eosin
ChIP: Chromatin immunoprecipitation
